# Remote glucose monitoring is feasible for patients and providers using a commercially available population health platform

**DOI:** 10.3389/fendo.2023.1063290

**Published:** 2023-02-02

**Authors:** Stephanie S. Crossen, Crystal C. Romero, Carrie Lewis, Nicole S. Glaser

**Affiliations:** ^1^ Department of Pediatrics, University of California Davis, Sacramento, CA, United States; ^2^ Center for Healthcare Policy and Research, University of California Davis, Sacramento, CA, United States

**Keywords:** remote patient monitoring, type 1 diabetes, population health, telehealth, continuous glucose monitoring

## Abstract

**Objective:**

Remote patient monitoring (RPM) holds potential to enable more individualized and effective care for patients with type 1 diabetes (T1D), but requires population analytics to focus limited clinical resources on patients most in need. We explored the feasibility of RPM from patient and provider standpoints using a commercially available data analytic platform (*glooko* Population Health) among a cohort of youth with T1D.

**Study design:**

Patients aged 1-20 years with established T1D (≥12 months) and CGM use (≥3 months) were recruited to participate. Participants’ CGM devices were connected to the *glooko* app and linked to the research team’s *glooko* account during a one-month baseline period. This was followed by a six-month intervention period during which participants with >15% of glucose values >250 mg/dl or >5% of values <70 mg/dl each month were contacted with personalized diabetes management recommendations. Participants were surveyed about their experiences, and effects on glycemic control were estimated *via* change in glucose management indicator (GMI) generated from CGM data at baseline and completion. Changes in time spent within various glucose ranges were also evaluated, and all glycemic metrics were compared to a non-randomized control group *via* difference-in-difference regression, adjusting for baseline characteristics.

**Results:**

Remote data-sharing was successful for 36 of 39 participants (92%). Between 33%-66% of participants merited outreach each month, and clinician outreach required a median of 10 minutes per event. RPM was reported to be helpful by 94% of participants. RPM was associated with a GMI change of -0.25% (*P*=0.047) for the entire cohort, and stratified analysis revealed greatest treatment effects among participants with baseline GMI of 8.0-9.4% (GMI change of -0.68%, *P*=0.047; 19.84% reduction in time spent >250 mg/dl, *P*=0.005).

**Conclusions:**

This study demonstrates the feasibility of RPM for patients with T1D using a commercially available population health platform, and suggests that RPM with clinician-initiated outreach may be particularly beneficial for patients with suboptimal glycemic control at entry. However, larger randomized studies are needed to fully explore the glycemic impact of RPM.

**Clinical trial registration:**

https://clinicaltrials.gov/ct2/show/NCT04696640, identifier NCT04696640.

## 1 Introduction

Modern therapeutic technology for type 1 diabetes (T1D) creates a large volume of patient-generated glucose data, but providers are not routinely accessing this data between clinic encounters to adjust diabetes management (1). The availability of Bluetooth-enabled continuous glucose monitoring (CGM) and blood glucose monitoring (BGM) devices (2–4) as well as multiple diabetes data-sharing platforms (5–8) have made remote access to patient-generated glucose data possible. In addition, population-level analytics have been developed within commercially available diabetes data platforms (9) to rapidly identify which patients exhibit high-risk data patterns during a given timeframe. Remote monitoring enhanced by population analytics has the potential to facilitate more person-centered care and to improve health outcomes by enabling clinicians to provide the right care at the right time to each patient, rather than waiting for scheduled clinical encounters to assess and adjust treatment. Clinician-initiated outreach based on patient-generated health data (PGHD) also has the potential to improve health equity within a population by prioritizing attention to those with highest clinical need rather than to those who are proactive in requesting between-visit care.

The dramatic rise in use of telehealth for diabetes care during the COVID-19 pandemic has increased familiarity with telehealth technologies and data-sharing among providers (10) and patients (11) alike, and has generated interest in hybrid care models that blend in-person care with synchronous and asynchronous telehealth. Key questions now relate to how such hybrid care models can be optimized for individuals and for populations while promoting person-centered care and health equity, as well as how they can be delivered efficiently with limited healthcare resources. Remote patient monitoring (RPM) with population analytics is likely to be an important part of diabetes care in the future, but before this modality can be effectively applied, its feasibility and utility must first be optimized from the patient and provider standpoints. This study begins to address those questions by piloting RPM with population analytics among a cohort of patients with T1D and reporting on feasibility, patient satisfaction, provider time requirements, and effects on glycemic control over a six-month period.

## 2 Materials and methods

### 2.1 Recruitment and enrollment

Participants were recruited by phone and/or during scheduled office visits or video visits at the UC Davis Health (UCDH) Pediatric Diabetes Clinic. Inclusion criteria were 1) age 1-20 years, 2) diagnosis of type 1 diabetes with a duration of ≥12 months (to avoid capturing the “honeymoon period” for glycemic control that occurs shortly after diagnosis), and 3) use of a continuous glucose monitoring (CGM) device for ≥3 months (to avoid capturing any improvement in glycemic control that was the result of improved self-monitoring after CGM initiation). Because this project utilized the Population Health analytic platform of glooko, patients were excluded from participation if their CGM devices could not be synced to glooko (e.g., Medtronic Guardian or Freestyle Libre). Written informed consent was obtained from participants ≥18 years of age, and from a parent or guardian for participants aged <18 years. Verbal assent was also obtained from minors 10 years and older. Verbal and written consent processes were conducted in English or Spanish depending on the preference of the participant.

At time of enrollment the research team collected demographic information from each participant’s electronic health record (EHR) for the purpose of characterizing our study population. Research team members assisted participants in setting up *glooko* accounts and establishing the necessary connections between their CGM devices and the *glooko* mobile application, as well as between participants’ *glooko* profiles and the research team’s *glooko* account. Successful linking of these devices and accounts enabled continuous, passive data-sharing from participants’ CGM devices to the research team’s *glooko* platform. Participants were informed at time of enrollment that the research team would not be evaluating their data in real-time (e.g., on a daily or hourly basis), and that they would need to reach out to the clinical diabetes team for assistance with any urgent diabetes-related concerns.

### 2.2 Baseline and intervention periods

During the one-month baseline period after enrollment, the research team tracked remote data-sharing by each participant and contacted individuals whose data was not transmitting consistently in order to identify and assist with any connectivity or software issues they encountered. Feasibility of remote glucose monitoring was defined by the proportion of enrolled participants who successfully established data-sharing by the conclusion of the baseline period. Participants who established remote data-sharing during this period advanced to the six-month intervention period.

During the six-month intervention period, the research team generated population analytic reports for the study cohort every month to identify participants with high frequencies of hypo- or hyperglycemia. The *glooko* Population Health platform allows providers to identify sub-cohorts of their patient populations meeting customizable glycemic criteria within the last 7, 14, 30, 90, or 180 days. Metrics can be specified for CGM and/or BGM data, including frequency of BGM measurements or CGM wear time, average glucose levels, and percentages of glucose readings above or below specific values. For this study, participants with CGM data demonstrating >15% of glucose values >250 mg/dl or >5% of values <70 mg/dl in the prior 30 days were identified each month and contacted by a pediatric endocrinologist (the principal investigator or PI) with individualized adjustments to their diabetes management plans. These thresholds for outreach were determined by analyzing baseline CGM data and identifying criteria which were met by 40-50% of the study population (to stay within expected time availability of the PI for outreach) but would also capture excessive hypoglycemia or severe hyperglycemia, balancing feasibility with the potential to improve glycemic control for those most at risk.

Recommendations provided by the investigator during RPM outreach were analogous to the care that would be offered if patients noticed their own concerning glucose trends and contacted the endocrinology team for assistance, except in this case the interaction was clinician-initiated. Advice was personalized and could include changes to insulin doses or pump settings, review of recommended self-care skills and routines for T1D, education about optimal use of diabetes technology, and/or emotional support for stressors related to T1D care if these were disclosed by the participant. Outreach took place either *via* asynchronous messaging in the EHR or *via* telephone for participants who did not have EHR messaging accounts, required language interpretation services, or did not read the initial EHR messages sent by the investigator. Language interpretation services were employed for all participants with non-English language preferences. During the intervention period, the duration and modality of each provider outreach event was recorded. At the end of the six-month intervention period, participants were surveyed regarding their experiences of and satisfaction with remote monitoring.

### 2.3 Glycemic control

This study was designed primarily to evaluate the feasibility and acceptability of RPM, and therefore was not powered to detect significant changes in glycemic control. However, given the availability of CGM data for all participants, we were able to generate preliminary estimates of the glycemic effects of RPM within our cohort. Glycemic management indicator (GMI) was used as the main indicator of glycemic control for study participants due to a high frequency of telehealth visits (at which hemoglobin A1c levels were not routinely measured) during the study period. GMI is a method of assessing glycemic control from CGM data, previously referred to as an “estimated A1c” (12). The GMI generated by *glooko* from participants’ CGM data over the prior 30 days was recorded at baseline and at study completion. In addition, time spent in various glucose ranges over the prior 30 days was recorded for participants at baseline and study completion, and changes in these metrics were analyzed along with changes in the glycemia risk index (GRI), which is a recently developed CGM-based metric that closely correlates with clinician assessment of clinical risk (13).

To isolate the effects of the intervention from any expected glycemic changes in its absence, we employed a non-randomized control group for this analysis. The research team made note of patients who met all enrollment criteria and had clinic appointments during the enrollment period but were not contacted for study recruitment due to limited research staff availability. Among these, a subset was already sharing CGM data to the UCDH Pediatric Diabetes clinic *via* the Dexcom Clarity data platform. We collected limited EHR data on these individuals – including demographic data and diabetes technology use – as well as the CGM data that these patients had voluntarily shared *via* Dexcom Clarity. The effects of the intervention on glycemic control were evaluated as the average treatment effect on the treated (ATT), using difference-in-difference regression to compare changes for treatment and control groups, and to estimate how much higher or lower the designated outcomes for the treatment group would have been if they had not received the intervention. This ATT analysis was performed using a linear regression model with clustered, robust variance estimation and adjustment for baseline characteristics of sex, age, race, ethnicity, insulin pump use, and insurance type.

Due to our expectation that treatment effects would vary based on participants’ level of glycemic control at study entry, we also stratified our analysis by baseline GMI quartile. This was accomplished by adding baseline GMI quartile to the model as an interaction term with the time and exposure variables, allowing the direction of change across timepoints to vary between quartiles. All participants with available CGM data were included in the glycemic analyses regardless of whether or how much they received outreach during the intervention period, in accordance with intention-to-treat principles. All statistical analyses were performed using Stata/SE version 17.0 for Windows (14).

## 3 Results

**Figure 1:**
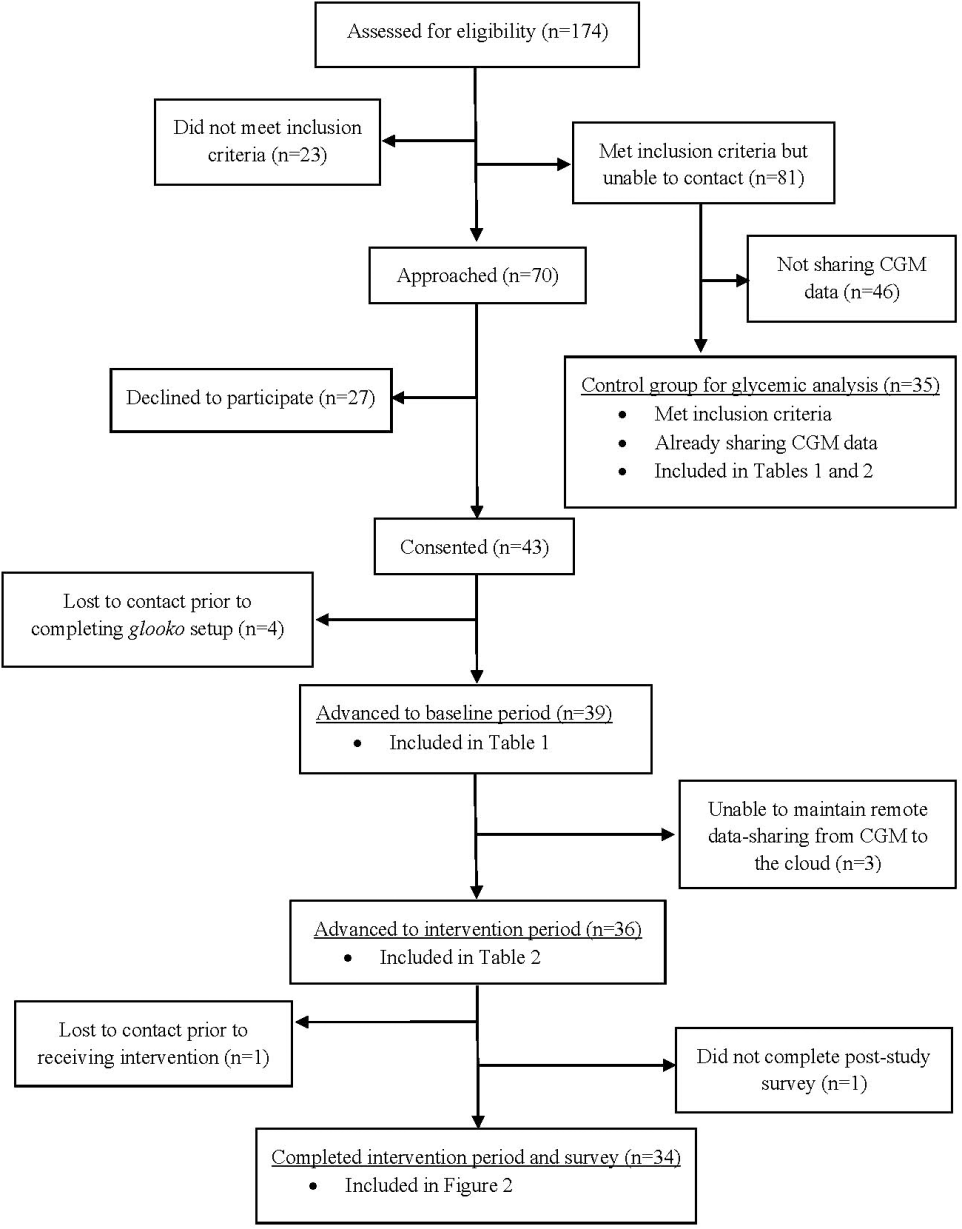
Study flow chart.

Recruitment took place between June and September of 2021, and is depicted in **Figure 1**. In total, 70 patients were approached about participation in the study. Of these, 43 (61%) consented to participate and 39 completed enrollment (establishing *glooko* profiles and linking their CGM devices). Baseline characteristics of these participants and of the control group are shown in **Table 1**. Both groups were approximately 40% publicly insured and 50% female, predominantly of White race and non-Hispanic/Latino ethnicity, and exhibited high rates of insulin pump use and relatively low mean baseline GMI. Age distribution differed slightly between the groups, with the treatment group containing a higher proportion of 10-15 year-olds and control group containing a higher proportion of children <10 years old. However, these differences did not reach statistical significance.

**Table 1:** Descriptive statistics by study group.

	Treatment (n=39)		Control (n=35)		*P* value^a^
**Age**					0.59
<10 years	9	(23.1%)	11	(31.4%)	
10 to 15 years	20	(51.3%)	14	(40.0%)	
>15 years	10	(25.6%)	10	(28.6%)	
**Female Sex**	21	(53.8%)	19	(54.3%)	0.97
**White Race**	27	(69.2%)	28	(80.0%)	0.29
**Hispanic/Latino**	7	(17.9%)	5	(14.3%)	0.75
**Public Insurance**	17	(43.6%)	15	(42.9%)	0.95
**Baseline pump use**	29	(74.4%)	24	(68.6%)	0.58
**Baseline GMI** ^b^	7.56%	[0.69]	7.50%	[0.73]	0.76

a Using χ^2^ test (categorical variables) or t-test (continuous variables).

b Reported as mean with standard deviation in brackets.

Three of the 39 enrolled participants were unable to establish consistent data-sharing due to trouble maintaining connections from their mobile devices’ *glooko* application to the cloud. The success rate for remote glucose monitoring was therefore 92%, and 36 participants advanced to the intervention period. During the intervention period, a median of 15.5 participants (43%) received outreach each month (range 12-24, 33-66%). Twenty percent of participants did not meet criteria for outreach at any time during the intervention period, while 26% received 1-2 contacts, 20% received 3-4 contacts, and 34% received 5-6 contacts. Clinician outreach required a median of 10 minutes per event (range 5-30), and a total of 105-275 minutes per month. Among those participants who received outreach, the median total duration of contact received throughout the study was 35 minutes (range 10-75). Overall, 61% of outreach events involved telephone contact (mean duration = 12.9 minutes, SD = 6.09), while 39% were conducted exclusively *via* EHR electronic messaging (mean duration = 5.9 minutes, SD = 1.92).

**Figure 2:**
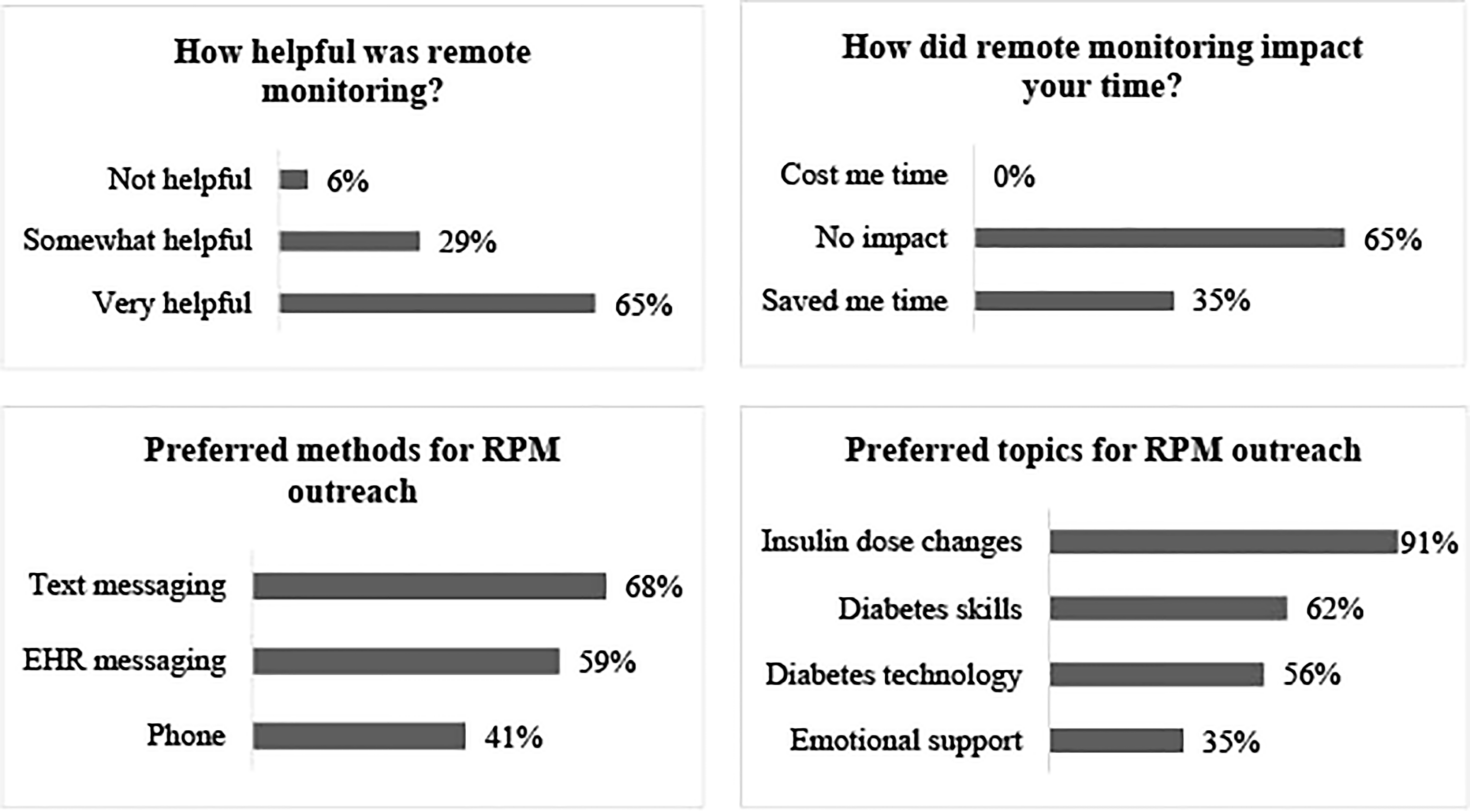
Participant survey results (n=34).

Survey responses were received from 34 participants who completed the intervention period, and are detailed in **Figure 2**. Sixty-five percent of respondents found remote glucose monitoring “very helpful” while 29% found it “somewhat helpful”. All participants responded that remote monitoring either saved them time or had no impact on their time. When asked about preferences for future remote glucose monitoring programs, participants favored communication from providers *via* text messaging or EHR messaging over telephone encounters. In addition, the vast majority preferred to receive advice about insulin dose changes, with lower proportions seeking advice about diabetes skills and behaviors, diabetes technology use, and emotional support related to diabetes care.

Glycemic analysis evaluating average treatment effect on the treated (**Table 2**) demonstrated a GMI change of -0.25% (95% CI -0.50 to -0.004, *P*=0.047) for the treatment group as a whole. However, stratified analysis by baseline GMI revealed more significant improvements among participants with baseline GMI of 8.0-9.4% (-0.68% change in GMI, *P*=0.047) or 7.6-7.9% (-0.55% change in GMI, *P*=0.066). Improvements in GMI for these two quartiles appeared to be driven primarily by reductions in time spent >250 mg/dl (-19.84%, *P*=0.005 and -11.35%, *P*=0.029, respectively), and resulted in a statistically significant improvement in GRI for the highest quartile (-19.74 change in GRI, *P*=0.026).

**Table 2:** Average treatment effect on the treated (ATT)^a^.

	∆ GMI (%)	∆ % Time Spent in Glucose Ranges					∆ GRI
		>250 mg/dl	181-250 mg/dl	70-180 mg/dl	54-69 mg/dl	<54 mg/dl	
**All - unadjusted**	-0.26						
	[-0.50, -0.20]						
	0.035						
**All - adjusted**^b^	-0.25						
	[-0.50, -0.004]						
	0.047						
**By baseline GMI quartile**^b^							
6.2-7.1%	0.01	-2.35	3.31	-3.11	1.31	0.52	3.59
	[0.25, 0.27]	[-10.45, 5.76]	[-1.42, 8.04]	[-13.5, 7.25]	[-0.14, 2.75]	[-1.04, 2.08]	[-14.84, 22.02]
	0.935	0.565	0.167	0.551	0.076	0.507	0.699
7.2-7.5%	0.06	-1.16	5.60	-4.64	-0.80	0.20	1.31
	[-0.11, 0.23]	[-5.18, 2.87]	[1.30, 9.90]	[-10.78, 1.49]	[-1.75, 0.15]	[-0.19, 0.59]	[-6.73, 9.35]
	0.472	0.569	0.011	0.135	0.098	0.315	0.746
7.6-7.9%	-0.55	-11.35	3.40	6.25	0.43	0.45	-13.05
	[-1.15, 0.04]	[-21.53, -1.17]	[-3.80, 10.60]	[-5.88, 18.37]	[-1.04, 1.90]	[-0.31, 1.22]	[-29.06, 2.96]
	0.066	0.029	0.349	0.308	0.563	0.242	0.109
8.0-9.4%	-0.68	-19.84	11.69	6.92	0.48	0.26	-19.74
	[-1.35, -0.01]	[-33.40, -6.29]	[2.47, 20.91]	[-5.35, 19.18]	[-0.37, 1.34]	[-0.12, 0.63]	[-37.08, -2.40]
	0.047	0.005	0.014	0.265	0.263	0.175	0.026

All results reported as mean, [95% CI], P value. GMI, glucose management indicator; GRI, glycemia risk index.

a Estimated via difference-in-difference analysis by clustered, robust variance estimation, linear regression model.

b Controlling for age, female, non-White, Hispanic/Latino, public insurance, and insulin pump use.

## 4 Discussion

### 4.1 Principal results

This pilot study of remote patient monitoring in a cohort of pediatric patients with T1D found that RPM was highly feasible and satisfactory for participants. Its findings also suggest RPM may be beneficial for glycemic management, particularly among participants with higher baseline glycemic levels (GMI ≥8%), who demonstrated a clinically and statistically significant GMI reduction over six months’ time. The fact that this subgroup’s glycemic improvement was achieved primarily *via* reduction in time spent >250 mg/dl – which was one of the metrics triggering clinician outreach in this study – suggests the RPM intervention was effective in addressing its target risk profile.

It is notable that these results were achieved with relatively infrequent clinician contact (monthly or less) and a median time of only 10 minutes per outreach event. The amount of clinician time required per month (1.75-4.6 hours total or 2.9-7.6 minutes per participant) for RPM in this study suggests that such an intervention could be feasibly scaled to larger populations. However, we expect that such time estimates are highly sensitive to the population’s baseline glycemic control, the specific outreach criteria, and the chosen method(s) of outreach, so these factors should be considered when attempting to extrapolate from our study’s time estimates to another RPM program. Importantly, our survey results indicate that most RPM recipients preferred asynchronous outreach over telephone contact, and study data demonstrated that the former also required less provider time than the latter. However, the research team found the utility of EHR messaging limited in our cohort by a high frequency of inactive accounts and unread messages, suggesting that future RPM programs would benefit from use of mobile text messaging instead.

### 4.2 Strengths and limitations

Strengths of our study include the use of a commercially available population health management tool, clinically prescribed CGM technology, and implementation of RPM with limited provider resources (one clinician), all of which replicate real world circumstances for many endocrinology practices. Our study population included adolescent and pre-adolescent aged participants, and a large proportion (>40%) with public insurance, which increases the generalizability of our findings. However, generalizability is limited by the fact that our cohort was majority non-Hispanic White, had unanimous CGM use and a high rate of insulin pump use, and demonstrated relatively good glycemic control at baseline.

Our small sample size was not powered to detect significant changes in glycemic control, as this study was designed primarily to explore feasibility, satisfaction, and efficiency measures. However, the fact that we did evaluate glycemic change *via* intention-to-treat regression analysis with comparison to a control group and adjustment for potential confounders is another strength. Based on the initial estimates of glycemic benefit from this pilot study, our research group is now conducting a larger, randomized study to evaluate the effects of monthly RPM outreach for patients with baseline hemoglobin A1c (HbA1c) ≥8%, including participants with and without CGM and insulin pump technology.

### 4.3 Comparison with prior work

Remote patient monitoring and population analytics within T1D care have been relatively understudied thus far due to their reliance on recently developed digital health technology and health informatics platforms. Improvements in glycemic control have been previously documented for adult populations enrolled in a commercial RPM program (15, 16), although these involved use of intermittent BGM (rather than CGM) and the majority of participants had type 2 rather than type 1 diabetes. The most pertinent existing literature to compare to our study comes from a single institution which has developed and deployed a homegrown population health platform to analyze CGM data remotely and identify pediatric T1D patients meeting high-risk criteria in order to enable outreach by clinical staff (17–19).

As in our study, this group employed RPM in a pediatric population with T1D all of whom used Dexcom CGM devices, although their population was newly diagnosed with T1D whereas ours was not (17–19). Other differences include that our study followed a smaller patient cohort (36 versus 89) for a shorter time (6 versus 12 months) with less frequent outreach (monthly versus weekly). Their RPM program resulted in a 8.8% increase in time-in-range and 0.58% decrease in HbA1c for participants after 12 months in comparison to a non-randomized, historical control group (17, 19), which is similar to the results we observed for the highest GMI quartile of our study population after 6 months. Their studies report RPM provider time as varying from 1.3 to 4.5 minutes per patient per week (17, 18). While this time investment is comparable to our study’s 2.9 to 7.6 minutes per patient per month, their program demonstrated greater efficiency due to more frequent review (1.3-4.5 versus 2.9-7.6 minutes per patient *per outreach period*). This greater efficiency may relate to the population health platforms used, to the efficiency of the clinicians involved, and/or to their exclusive use of electronic messaging and our study’s inclusion of telephone encounters, which tended to require more time. Participant satisfaction and perceived feasibility for their program have not been reported to our knowledge.

### 4.4 Context

Many questions remain about how to optimize the use of RPM with population analytics for diabetes patients and providers alike. Challenges for practices who seek to implement RPM programs include: 1) utilizing a single data platform for all patients’ diabetes devices, 2) determining which metrics should trigger clinician outreach, 3) building the necessary staffing and workflows to support this type of unscheduled care, and 4) ensuring financial *via*bility.

We are aware of one commercially available population analytic platform for diabetes data that is compatible with a majority of (but not all) commercially available diabetes devices (9), and one additional such platform that is in development (5). However, currently available platforms focus on CGM data without BGM or pump compatibility. Utilizing a population health platform that is not compatible with all diabetes devices risks excluding a subset of the patient population from RPM services. While CGM is now recommended for all individuals with insulin-requiring diabetes (20), significant gaps continue to exist in access and use for individuals with T1D, particularly among low-income and historically marginalized communities (21). In addition, many adults and children with type 2 diabetes do not have access to CGM devices but could potentially benefit from RPM care. Diabetes data platforms that offer population analytics should continue to expand their compatibility with BGM, CGM, and insulin pump devices moving forward in order to maximize access to RPM for all people with diabetes.

The metrics used to trigger clinician outreach in an RPM program will depend on staffing, anticipated frequency of review, and clinical goals at a population level. These three factors are highly interdependent. For example, if the clinical goal is to assist patients who are not self-monitoring closely enough, metrics should focus on frequency of CGM or BGM data and outreach should be frequent (perhaps weekly) to promote stepwise behavior change. In contrast, an RPM program aiming to reduce hypoglycemic events would choose metrics focused on frequency of glucose values <70 mg/dl or <54 mg/dl and might structure outreach on a monthly or twice monthly basis in order to capture persistent patient-level trends that warrant attention. The criteria for outreach (e.g., <3 BGM values per day, >5% CGM time <70 mg/dl) should then be selected in light of staff availability and the anticipated data profile of the target population, but may need to change over time to maintain a feasible workload. For example, in the current study, outreach was initially planned for all individuals not meeting published CGM targets (22), but preliminary review of CGM data for our cohort indicated that this would necessitate contacting the majority of the cohort every month, which would require more clinician time than available. Therefore, less stringent thresholds for outreach were adopted with a goal of targeting participants experiencing the most frequent severe hyperglycemia or hypoglycemia.

By tailoring the outreach threshold(s) to the patient population and provider time available, RPM programs are more likely to be successful. Starting with a targeted goal allows practices to build RPM workflows on a pilot basis that can later be scaled up to support larger population-wide programs. Pilot programs also enable practices to explore financial *via*bility (e.g., reimbursement for remote monitoring CPT codes or capitated agreements with payors), which in turn may determine how much provider time can be diverted from in-person or telehealth appointments to provide RPM care.

## 5 Conclusions

The ability to access patient-generated glucose data remotely has opened the door for new models of T1D care that are more individualized in terms of the frequency and timing of outreach. Such models have the potential to be more responsive to patient needs, to enable more efficient and effective use of clinician time and practice resources, and thus to improve not only individual health outcomes but also health equity within T1D patient populations. In comparison, current care models are prescriptive (scheduled quarterly visits) and tend to prioritize between-visit care for patients with the highest health literacy and self-efficacy (those who initiate supplemental telephone and EHR messaging encounters with their clinicians).

Patient and provider openness to telehealth care modalities – including remote monitoring of PGHD and asynchronous electronic interaction – has increased with the rapid uptick in telehealth use necessitated by the COVID-19 pandemic (10, 11). However, shifting from a traditional model of T1D care that is prescriptive and reactive to a new one that is proactive and involves ongoing triage of PGHD, determination of appropriate outreach thresholds, and deployment of clinical staff to meet the ever-changing needs of a given population is a complex challenge. Practices will have to create new workflows, rethink staffing ratios, and consider reimbursement strategies and patient education approaches that support the new methods and cadence of this type of T1D care delivery. Pilot projects can lay the groundwork for such processes by exploring data platforms, care algorithms, and outreach strategies for remote glucose monitoring with attention to resulting patient- and provider-centered outcomes. This pilot study provides one such example, which can be used to inform future RPM efforts. This study and others of its type are essential so that practices can design RPM programs which efficiently provide individualized T1D care without over-burdening a limited supply of clinicians.

## Data availability statement

The raw data supporting the conclusions of this article will be made available by the authors, without undue reservation.

## Ethics statement

The studies involving human participants were reviewed and approved by the UC Davis Health Institutional Review Board. Written informed consent to participate in this study was provided by the participants’ legal guardian/next of kin.

## Author contributions

SC conceptualized and designed the study, performed the intervention, collected, analyzed, and interpreted the data, and drafted and critically revised the manuscript. CR recruited the participants, collected, analyzed, and interpreted the data, and critically revised the manuscript. CL analyzed and interpreted the data, and critically revised the manuscript. NG assisted with study design, interpreted the data, and critically revised the manuscript. All authors contributed to the article and approved the submitted version.
